# International Dental Federation: First General Meeting, Held at Cambridge, England, 1901

**Published:** 1901-12

**Authors:** 


### INTERNATIONAL DENTAL FEDERATION: FIRST GENERAL MEETING, HELD AT CAMBRIDGE, ENGLAND, 1901.

(Continued from page 796.)

The President said that after the remarkable speech of Sir Michael Foster he could not hope to say much, because his knowledge of English was very limited. He was almost impelled to close his manuscript and sit down, as there appeared to be nothing more to say. He had come to the town of Cambridge and to its university not only to place himself under the instruction of great philosophers, but to feel the influence of the education which had been going on there for centuries. Sir Michael Foster had spoken and advised on a question which divides the dentists throughout the whole world, and Sir Michael had said what was to be said better than any one else had ever said it before. But, as he had prepared a speech, he suppcsed that he must say something, and he claimed their indulgence.

The President then delivered the following

### INAUGURAL ADDRESS.

Mr. Chairman and Gentlemen,—Last year, the twelve hundred dentists who attended the Third International Dental Congress, which was held at the time of the Paris Exposition, decided to preserve the professional organization created in view of that Congress by organizing the International Dental Federation, with its Executive Council and an International Commission of Education. The dentists wanted to form a universal union of the nature of that formed several months afterwards in the case of the *sociétés savantes* while waiting for the time when diplomats and governments should realize this union in all the branches of human activity, making the latter a compact entity. According to this decision, a meeting was held in Paris immediately after the Congress.

This year we have come to England to hold our second meeting. The welcome that has been tendered us, the prominent men that have taken part in our discussions, and the first results of our work show that the organization newly created by the Paris Congress supplies a necessity of our epoch, and that it will produce useful results for the advancement of the science of odontology. I have maintained that the meetings of the International Dental Federation attract the choice men of our profession. And indeed they are choice men,—those who do not hesitate to leave their families, their occupations, and their countries to discuss topics not of direct or immediate interest to them, but that constitute the problem of the education of their successors. Our *confrères* will certainly ratify the statements just made when they see the list of practitioners that have attended our meetings, representing sixteen of the most important countries of Europe and America.

As far as our labors are concerned up to the present, they have consisted mainly of an exchange of ideas in view of organizing and devising a plan of work for our future meetings. The more complex and important a body is, the longer must be its period of organization.

The birth of the new element of professional progress took place last year in the midst of great ceremonies, and in the presence of the most authoritative representatives of the dental profession of the world, in a meeting presided over by one of the greatest *savants* of the University of Paris, Professor Gariel, the delegate of the government. It has been the desire of the Executive Council that the second meeting, which constitutes for our International Federation a sort of scientific baptism, should take place with the same ceremonials, and for this purpose it has requested the eminent vice-chancellor of the University of Cambridge to stand as its godfather.

The Executive Council has honored me with the delicate mission of indicating the purpose that we are working for. Pardon me, gentlemen, if I confess to a feeling of timidity in having to address so distinguished an audience, in view of the knowledge and talent properly required for such a task. I shall, however, endeavor to fill any lack in those two factors by contributing all the good-will of which I am capable.

Our work is one in which we have to deal constantly with difficult problems. In the first place we have the diversity of languages; this, however, is an obstacle easily overcome. There is another one of greater magnitude, brought about by the character of the different countries in which we live, by the difference in the legal conditions of their organization, and especially in their degree of evolution,—evolution which is in some slight degree the cause of our diversity of opinions. We must make great efforts and many mutual concessions in order to harmonize our national conceptions into the international plan which it is our wish should emanate from our discussions. A certain amount of work is also necessary, in order to set forth this programme in such a way as to be understood and accepted by every one of us.

It seems to me that it is a daring act for me, the modest representative of a science as yet new, to stand here in this old university, before such distinguished professors, and talk to you on education,—that is, on one of the highest problems, even though it be limited to the training of young dental surgeons. Therefore I have aimed to shelter my affirmations under the authority of men whose names and writings are highly valued in this connection. Two thoughts that I have borrowed from Michelet have encouraged me. One is that education is the first, the second, and the third part of politics,—that is, its pure essence; and the other is that in an advanced society teaching ought to be the function of almost every one.

While working at the preparation of this paper, my mind was impressed with the importance—for the future of humanity—of an Anglo-French combination. And so I consulted alternately the French and English philosophers. I went from Michelet to Herbert Spencer to Stuart Mill, and from Roger Bacon to Descartes, in order to borrow from them the general principles which must guide us in the preparation of a national programme of instruction in the midst of the contradictory ideas which are contending for the direction of public education. It has been through the inspiration of their principles that we are able to know the value of every science which is to be part of this programme, and adopt a method of appreciation of their worth; and also in order to know the means of differentiating between studies having an intrinsic value and those having a merely conventional one, and between those that are valuable from the view-point of knowledge and those that are important from the stand-point of education and discipline.

We shall then understand better the difficulties of education in general, with the harm that can be done to the mind by the studies that may enter into the formation of a programme or by the faulty order in which they are taught; also the necessity of proceeding from the concrete to the abstract rather than from the abstract to the concrete. It will be easier to understand the necessity of comparing these general principles of education with those that inspired the curricula of our schools, prepared empirically and according to the needs of the respective countries.

Among the general notions capable of directing us in our work, and that can be transformed into accurate proportions applicable to the teachings with which we are concerned, I will quote the following paragraph from the admirable work on Education by Herbert Spencer: "One of the conclusions at which we arrive is that in every branch of knowledge we must proceed from the empirical to the rational. A leading fact in human progress is that every science is evolved out of its corresponding art. It results from the necessity we are under, both individually and as a race, of reaching the abstract by way of the concrete; that there must be practice and accruing experience, with its empirical generalizations, before there can be science. Science is organized knowledge, and before knowledge can be organized some of it must first be possessed. Every study, therefore, should have a purely experimental introduction, and only after an ample fund of observations has been accumulated should reasoning begin."

It is curious to observe that this proposition of the English philosopher can be easily applied to the organization of dental education, such as has been very well understood by the different nations, especially by the United States, England, and France. In fact, in the English schools the apprenticeship of dental prosthesis precedes the special scientific and medical studies, which, in the evolution of the profession, is according to the historical evolution indicated by Herbert Spencer, and according to the principle of the subordination of the abstract to the concrete. We will endeavor to inspire ourselves with the principles of those great thinkers who have studied education in general, that we may apply them to our particular branch of education, whose purpose is to form good odontologists.

It is not an indifferent question, for the state and for the people at large, that odontologists should receive a rational education in proportion with the progress of their special science,—an education capable of developing their qualities to their maximum.

It is not necessary that I should say anything to you upon the utility of the dental system for the preservation of health. At an epoch not very remote from this good teeth were an indispensable requirement of the soldier, who, however, had to open cartridges with his teeth. Progress in the military art has caused the disappearance of this practice. The state should by no means, just for this reason, be indifferent concerning the organs under our supervision. The teeth are always necessary for the development of the child, as well as for the conservation of health in the adult. Dentistry should hence be in evidence in the school, in the army, and in all aggregations of human beings as an important part of hygiene.

Likewise, it seems unnecessary that I should call the attention of dentists to the importance, for their dignity and for the position that they should occupy in the state and in the public estimation, that they should receive a complete and rational education, which would permit them to render to their fellow-citizens all the services within their province. A good education for the dentist is for the state a question of public interest, as it is for the dentists themselves a question of professional advantage. This for a long time has been perfectly well comprehended by our *confrères,* as can be seen by the papers on these questions that were read at our last professional meetings.

From the foregoing, it can be seen that the international union that we are endeavoring to bring about is useful and desirable, but the obstacles to which I have already referred, obstacles with regard to the laws, customs, traditions, the routine, the prejudices of every country, are numerous. There are others inherent to the conditions of evolution of odontological science, and to which I shall refer only in brief. Odontological science, from the view-point of special science, is relatively a new one, at least it has been recognized as such only in recent times. Recent historical works show that in all refined communities, in Egypt, in Greece, in Rome, the dentist existed in remote times as a special practitioner.

At the beginning, when medicine was closely connected with the priesthood, the priest, and later on the physician, were able to treat the disorders of the teeth as well as those of the other organs of the body, but soon a different kind of treatment became necessary,—the prosthetic or restorative one, involving a mechanical art with which the physician was not familiarized and to which he was not sympathetic, according to the expression used by Dr. Kirk. It is this prosthetic art that gave birth to the prosthetic dentist.

The evolution of odontological science has taken place not without a certain amount of antagonism between these two kinds of practitioners; the physician, on the one hand, occupying himself with the purely medical phase of the diseases of the mouth, and the specialist of dentistry of the prosthetic phase, being a simple artisan at first, becoming later on the surgeon-dentist, having added gradually to his technical training in order to reach that step,—the study of the medical branches directly applicable to the needs of his *clientèle.*

As Dr. Kirk has very wisely said, it is he who can be looked upon as the departing-point in professional dental evolution in all countries. It is by him and for him that this evolution has taken place. It is for him that in 1700 special laws were framed in France; it is for him that later on, in 1726, Fauchard, the father of modern dentistry, wrote the first special and complete work on dentistry. It is for him that in 1838, in Baltimore, Harris and his friends founded the first dental school, separately from the schools of medicine, after an interesting declaration of independence, which can be considered with Fauchard's book as the *acte de naissance* and the scientific basis of odontological autonomy.

A certain number of dentists, holders of the medical degree, considering that medicine and dentistry should be indissolubly ligated, did not adapt themselves to the plan that dentistry should be taught and practised as a specialty of medicine. And, as Dr. Kirk says, this opinion persists in spite of the success obtained by the dental schools in all the countries of the world, and also notwithstanding the fact that the separation between medicine and dentistry is becoming more accentuated from the stand-points of both education and practice. Some countries of Europe have followed this doctrine, and have arranged accordingly education and practice.

It is understood that, according to whether one or the other principle is accepted, the solution differs as far as education is concerned. In fact, if odontology is considered as a simple medical specialty, as ophthalmology, laryngology, and gynæcology, it suffices that the student should first conclude his medical education in a medical school, and then, if he thought it necessary, he would go for a few months to a dental school in order to familiarize himself with the technique of dentistry that he did not learn in the hospitals. In the second case the student enters from the beginning the dental school, where he completes his entire education, just as the student in pharmacy studies his profession in a school of pharmacy, with the addition, if necessary, of a few special courses at the school of medicine or at the hospital.

In order to cause the disappearance of the antagonism of these conceptions, and to dissipate the difficulties that they oppose to the progress of our teaching, it is necessary, according to Herbert Spencer, to bring to light the facts which have been accumulated in sixty years of teaching in the countries where dental education has been organized in an empirical manner,—that is, according to the needs and necessities of the time. For this purpose we have to make appeal to impartial statistics. This is the first part of the work of the International Commission of Education. This introductory work is important from the stand-point of the direction in which dental education should go, and also from that of the position that this teaching should occupy in the universities. But this question is not the only one that requires our attention; there are others, of secondary importance, it is true, but nevertheless questions which the representatives of both doctrines could discuss to advantage and agree upon. Among these we should include the problem of the necessary preliminary education for the dentist.

On this question we also see the reappearance of rivalries of the classical and scientific education with regard to the utility of the mechanical and preliminary professional education, such as is given in the United States in the manual training-schools, in France in the École Diderot, as adopted by the Paris Congress, and such as we see it in London in the Institute of Technology as organized by our friend Cunningham. We will also mention the question of the extent of the scientific programmes, medical or technical, theoretical or practical.

In order to determine exactly the studies which should enter into the programme, we must first of all determine the duration of these studies. It is evident that when the time shall come for the discussion of the quantity of medical sciences and of mechanical art that should compose the programme of the future dental surgeon we will again find the partisans of the two opposite principles. But then one factor interposes itself like an arbitrator; I mean the duration of the course,—the number of hours that could be reasonably consecrated to dentistry. "Had we time," says Herbert Spencer, "to master all subjects, we need not be particular." To quote the old song:

> "Could a man be secure
> That his days would endure,
> As of old, for a thousand long years,
> What things might he know!
> What deeds might he do!
> And all without hurry or care."

But "we that have but span-long lives" must ever bear in mind our limited time for acquisition. It is superfluous to declare that we must limit ourselves to the useful, to the essential, to the indispensable. "It is not necessary," says Descartes, "that the honest man should have read all books, neither is it necessary that he should have learned everything that is taught in the schools. Moreover, it would mean a mistake in his education if he had consecrated too much time to the study of letters; there are many other things to be done in life." Hence the time that it is possible to devote to study will be co-arbitrator with another factor,—the number and nature of the operations which the dentist is called upon to perform at the present time in the branches of operative and prosthetic dentistry and anæsthesia.

Lastly, the results obtained in the different educational centres, with the different systems in vogue in America, England, France, Switzerland, Austria, Germany, and Russia, will also enter in this account. This is why the active collaboration of the men of all countries is necessary. Then we will see that by placing ourselves on an international stand-point all our discussions will lose some of their acerbity and intensity, and we will get rid of the irritating questions which often refer to minor considerations—mere words or purely local designations—rather than to real division.

By elevating the discussion to a philosophical standard, we shall agree that odontology is a science which tends to the preservation of man; it is a biological science. Hence it is perfectly possible to conceive, according to Professor Eliot, president of Harvard University, recently quoted by Dr. Kirk, a new university where the teaching of biological sciences would be established on a broad basis, so that all students, according to the purpose of their studies in relation to the profession chosen,—so that practitioners or savants, physicians or dentists, should be able to take up the fundamental knowledge which they would require while following the study of a specialty not taking up more than about four years, and to conclude by obtaining the final diploma of doctor in this specialty.

Under such conditions dentistry, says Dr. Kirk, would have a place in medical education or, better, in the university in proportion to the needs of its practice, and the antagonism to which we have referred would not exist.

But, no matter what the future reserved to this proposition is, in the mean time the discussions of the International Commission of Education will contribute to advance in our several countries the question of the position of the dental surgeon and of his education. We want to work for the benefit of our successors, and prepare the best programme of intellectual, moral, and physical education. Besides, in this work there is a thought capable of giving us much satisfaction,—the thought of love of humanity; and love, according to Auguste Comte, is the secret of human nature, the secret of the world.

We have applied to this work the principles of thinkers and of philosophers of whom humanity feels honored. By the welcome that has been tendered us we are assured of the support of the savants of this university. This is our encouragement, and also our reward.

This work is new; it will have to be developed by others, and, as stated by that great man who went through this university,—Roger Bacon,—"Many will pass and science will grow."

*Sir Michael Foster.*—I am sure we have all listened with very great pleasure to the admirable address of our president, and did that great trouble, time, allow we might proceed to discuss some of the points that he has raised. But it is my duty—not only as deputy vice-chancellor, but as professor of physiology—to remind you that in a very short time you will be called upon to perform, according to your programme, certain physiological operations in which the teeth play an important part. I think we shall be consulting our interests if we defer further discussion until this afternoon, and now gently wend our way to the Combination-Room of Trinity College, where the vice-chancellor has provided for you something to eat.

LUNCHEON.

At the invitation of the vice-chancellor of the university, and with the kind consent of the Fellows of Trinity College, the delegates were entertained at luncheon in the Fellows' Combination-Room, Trinity College. Sir Michael Foster again presided, and, with the Regius Professor of Medicine and Dr. Cunningham as "croupiers," at the conclusion of the luncheon said:

The autocrat of all the dentists, Cunningham, has issued a decree that there shall be no speeches at this luncheon. I understand you are going to have as many as you want this evening at the banquet, but we are to have none here. Still, I think you would like me to convey on your behalf some words to the person who is the real vice-chancellor of this university. I am only for the time being the deputy vice-chancellor; the real vice-chancellor, I hope, is fishing in Scotland, recruiting his health, which has been impaired by his many labors during the session. But I may say that I will convey to him on the part of you all your best thanks for the opportunity you have had in meeting in this old university, and also—may I add?—for the excellent repast, which I am sure he ordered in fear and trembling, knowing how severe critics you dentists were in everything that concerns the mouth.

At the instance of Dr. Godon, the healths of the vice-chancellor and of Sir Michael Foster were drunk with acclamation, Dr. Godon remarking that the Federation had never been, and never would be, better treated than it had been that day.

CONFERENCE.

In the afternoon a conference was held in the Trinity College Hall, over which Sir Michael Foster presided.

The following address on "Dental Education" was read by Dr. Joseph Griffiths (University Reader in Surgery):

Sir Michael Foster and Gentlemen,—I feel this is an occasion for introducing what I am about to say with apologies, because neither am I a dentist nor indeed do I know, except in very general terms, anything of the education of a dentist. But, as I have been asked to speak upon the subject now under consideration, I beg you will grant me that indulgence which you, tried by meetings and speeches during the last week, must be pretty well accustomed to exercise, and which I trust you will freely bestow upon me and the matter of my remarks. As, however, I represent the sister art of Surgery in this university, and am engaged in the teaching of the art, as well as the science upon which the art is built up, I may be allowed to have a small say in the matter of the education of the dentist. This subject is not new to me, thanks to Dr. Cunningham, who has on many occasions brought it before me for discussion on the main principles which should guide in the bringing up of the dentist. As I have said above, I know nothing of the work of the dentist except in a general sort of way, which I gather on the occasions when I seek his advice and aid, and all I can hope to bring to the discussion of this interesting subject is the *point of view* of the surgeon, who is in one sense the father of the dentist, he being even now capable by law of undertaking, if he chooses, the practice of dentistry.

Now, I believe I am correct in making the following statement, —that the dentists are divided among themselves as to the best means to adopt whereby they themselves can be best educated; and, broadly speaking, they are divided into two sections. To both sections, however, the desire to produce the best dentist is common, and each section naturally thinks it has found the right way. So far as I am able to gather and understand, this difference between the two sections may be expressed in the following manner: One section desires that every dentist shall be trained as a medical man is, and then take up dentistry; whereas the other section desires that a dentist shall be trained to his own profession from first to last. (Before we proceed any farther, I think it would be well for me to state that all I am about to say applies to the average, and not to the exceptional, dentist.) To emphasize this proposition, let us put it thus: One section desires dentists to be qualified medical men who have, as it were, taken up dentistry as an after-thought, and the other wishes for a dentist from start to finish. According to the former, the man would be given a general medical education, and it can be estimated at nothing more, to base his future practice of dentistry upon; whereas, according to the latter, he would be given an education upon which his future work directly depends.

Is the education of a dentist to be that of a medical man with dentistry added on, or is it to be designed to meet his own requirements? is the question of the hour.

To help in the solution of this interesting problem, a brief comparison between the training of the medical man and of the dentist may not be out of place. In the case of a medical man the first half of his educational career is spent in gaining a complete knowledge of the normal man, and he takes biology, chemistry, and physics as introductory subjects to anatomy and physiology. This is done in order to give him a better understanding of the structure of the body in detail and of the functions of its several organs and tissues. The second half is spent in acquiring all that is known of morbid changes and abnormal functions, and in a training in the physical examination of any and every part of the human frame. In the earlier half, then, he is trained in methods adopted in the different subjects for eliciting knowledge, and in the second he is directed to employ the methods with which he is already familiar to determine as far as possible the physical condition of any or every part of the body.

On the other hand, in the case of the dentist the first period is spent in acquiring the knowledge of the nature and of the mechanical properties of certain materials and in the training to perform accurate work, which must be done, so I understand, to a nicety,—a training similar to that of a mechanician. In the second period he is directed to acquire a general knowledge of the structure of the body and of the functions of its several parts; a minute acquaintance with the teeth and the jaws, and of the diseases they are liable to; with the application of the methods, already familiar to him, of dealing with the teeth in their morbid states.

Such I believe to be a fair general statement regarding the training at the present time of a medical man and of a dentist. Let us contrast the requirements of these two. The medical man requires a knowledge of the minute structure and of the functions of the whole body, but the dentist only a knowledge of the minute structure of the teeth and of the jaws, and a general idea of the rest of the human frame. A medical man requires only a general, but sound, idea of mechanical work, but the dentist a thorough knowledge of it, so that he may be able to perform his work with accuracy. A medical man requires a detailed knowledge of all diseased processes and their known causes, but the dentist a particular knowledge of morbid processes as seen in the teeth and jaws, and only a general idea of the morbid processes observed in the remainder of the body. Such a review brings out pretty clearly that the educational career of a medical man does not coincide with that of the dentist except in a few particulars.

Even in anatomy and physiology, in which their work comes nearest together, the dental student requires that which will give him a sound understanding of the teeth and their connection with, and relation to, the remainder of the body, whereas the medical student should be familiar with the whole body. Of course, the more a dentist knows of the human body or of any other kindred subject the better he will be equipped generally, but not necessarily better furnished for the work of his own profession.

And if we go farther and compare the surgeon with the dentist, we shall find that their work differs in a material degree. The dentist must possess many of the qualities that go to make a surgeon; he should have a quick perception, a keen insight, a sensitive touch, and be ever ready to act. But the skill of the dentist is largely, if not entirely, the result of that training in the mechanical department, so to speak, whereas the skill of the surgeon depends less upon mechanical training than upon accurate judgment to do enough and no more,—for in hardly any operation is it necessary for him to make a physicist's measurements and to adhere to them. Mechanical training has indeed been neglected in the education of a surgeon, and hence it is that we often deplore the mechanical knowledge and the reasoning built upon its deficiency as displayed even by surgeons of repute. This has been neglected, I imagine, because that skill born of judgment has been estimated so highly. Now, with the dentist it is just the reverse, for who can conceive a dentist who is ignorant of mechanical work?—but one without judgment may perhaps be occasionally met with. Thus the surgeon is at one end of the scale and the dentist is at the other, and doubtless it would be a good thing to improve them both, but in contrary directions.

Enough has, I hope, been said to point out that to make a dentist his training should be so arranged as to bring out his fitness for the work before him. A mechanical training of the best kind is essential to him, and must form the basis of his future work. In addition he requires a knowledge of the minute structure and of the function of the teeth, the material upon which he will have to bring his best mechanical skill to bear. I would therefore strongly urge you not to imitate the education of a medical student, but to continue on the lines which will train a dentist for his own profession from first to last, and to have a single purpose in view and to endeavor to obtain a definite result. Do not try to make a medical man a dentist, but let a dentist start and finish as such.

Can this education of a dentist be carried on side by side with that of the medical man? is the question of practical importance. I would unhesitatingly answer, No. The anatomist may train either, but he cannot train both together without giving one much more than he requires and not paying enough attention to the other. It is much the same with physiology. Therefore I say their courses should be separate, and so arranged as to serve the right end. In physics and chemistry the same training might serve; in study of diseases, No.

Is such a course of study proper for a university to undertake? In my humble opinion it is and should be, for the work of the dentist is as honorable and as worthy of respect as that of any of the older professions, and I trust that the newer universities will take this line and have an avenue for dental students to obtain a university degree side by side with the medical student. But I also trust the authorities will let dentistry and medicine be free to develop along those lines which each finds best suited for its own progress.

Although dentistry was once an intimate part of the medical art, it can hardly be so again, for its evolution has been so complete that it now forms a distinct and separate division of the art of healing. It is, I venture to think, a child of the old stock destined to continue an independent existence and to work out its own salvation.

At the conclusion of the address, and before the opening of the discussion, Mr. W. B. Patterson, the honorary secretary of the British Dental Association, entered the room, and was heartily welcomed by Sir Michael Foster.

*Dr. Brophy* (Chicago) said that years ago he held views quite contrary to those he held to-day, but in the light of advancing education and the development of dentistry he had been forced to accept ideas that were formerly not agreeable to him. In the United

States dentistry had its birth as a separate and distinct school of training; it was not from choice, but because the medical profession refused to give dentistry a place in their curriculum. At that time it was regretted, because it was wished that dentistry should be a part of the parent medical profession. But, independent schools having been founded, such branches of medicine were taught as the founders felt were necessary. The schools had now added, from time to time, departments until one who was not acquainted with all the medical curriculum might readily be led to believe that they were schools of medicine. It was recognized that in Europe the conditions were quite different from those in America. It was felt that it was quite impossible to make changes in this country, and in many instances it was quite impossible to make changes in America. The question, therefore, was how to prepare a man to do his work. He had the pleasure of sitting by the side of Sir Michael Foster at luncheon, and he put to him the question. He had a boy nineteen years of age, just ready to enter the university; should he be prepared by a long four years' course in the university and then entered at the school of medicine for another four years' course or not? The answer was, "I would prepare him for his life's work. A student of medicine should devote his attention to the study of physics and chemistry and languages." Had he learned nothing more in his trip to Europe, he would have been fully compensated by that answer. He quite agreed with every word contained in the paper. The man who started out in life to prepare himself for any particular calling must have in mind that calling from the beginning to the end.

*Dr. Sims Woodhead* (Professor of Pathology, Cambridge) said that every man preparing for his life's work underwent a certain amount of general training, a training to fit him for the specific work he had to undertake later; and he could not help thinking that perhaps the preliminary years of the dental student and medical student might at any rate run on certain parallel lines. An attempt was made, how far successfully it was difficult to say, in the subject he had to deal with to give the medical student, as soon as he came from his study of anatomy and physiology, some inkling of the general processes of disease. The student specialized in the direction of special pathology, diseases of the nervous system, diseases of the kidney, and so on; but before he took this up it was absolutely necessary he should have a good solid foundation of the general processes of disease. That being the case, he could not help thinking it might be somewhat dangerous to begin to specialize at too early a stage, and whether it would not be better to study at any rate the general physiological and pathological processes, in order that in studying the disease of the special parts to which attention was to be devoted one might go to the very foundation straight away. From that point of view he put in a plea for some common ground in the earlier part of the dentist's professional life. He recognized how much dental surgeons had contributed to the subject of bacteriology. In fact, the earliest experiments in bacteriology were carried out on material taken from the teeth. He quite agreed with dentists specializing in the later stages of their course and not attempting to be medical men or surgeons, because they wished to treat a patient for special diseases of special organs, and it was their duty to know far more about those than any medical man or surgeon possibly could know. Dentists were experts, and therefore required an expert training. Even the surgeon found it necessary to specialize, and anything outside his own work he handed over to a colleague. In that light he hoped no attempt would be made to make the dental qualification a medical qualification, but that it would be made something far better for the purpose than any medical or surgical qualification. The dental surgeon was a man specializing in a certain direction, who had built up his profession on a good sound foundation of general physiological and pathological knowledge.

*Professor Hessé* (Leipzig) considered that the preparation for the dental profession was so different in different countries that it was almost impossible to fix a rule or a standard for the preparation in all countries. It was necessary to be guided by the point in view,—namely, the development of the art or profession which it was intended to practise, and the training should be such as was best fitted to secure that end.

*Dr. Aguilar* (Madrid) said he was deeply interested in the proceedings of the Federation, because he was the first to feel the necessity of such deliberations. It was only a few months ago that in Spain the law for establishing a dental department in the University of Madrid was passed. Prior to that there was no dental teaching except what was given privately. He had the honor of being appointed to the Chair of Dentology in the University of Madrid, and when he was called upon to propose the curriculum of studies he felt the necessity of learning the opinion of learned men,—and that opinion could not be better gained than through the Federation now assembled in Cambridge. He felt himself fully compensated and over-compensated for the trouble of attending the meeting. He had no authority to express an opinion of his own, and he would only place in the hands of the chairman the following proposition:

"That five members be appointed to propose resolutions on the following questions and report at the next meeting:

"(1) What preliminary studies should be required for the admittance of students into the dental colleges?

"(2) What are the technical, theoretical, and manual studies the student should pass through before being allowed to practise dentistry?

"(3) What part of the studies taught in the medical colleges should be followed by dental students?

"(4) What are the most reasonable titles to be applied to the persons who practise the therapeutic and prosthetic treatment of the diseases of the teeth and mouth?"

On the motion of Dr. George Cunningham, seconded by Dr. Brophy, the resolutions were referred to the Committee of Education, with full power to act.

*Dr. Kirk.*—It was said some years ago by a gentleman who resides not so many miles from this spot that the "evil that men do lives after them; the good is oft interred with their bones." But in what has taken place at this conference it seems to me we have at least one instance where the reverse of that proposition is true. If I have been able to correctly interpret the *motif* of the eloquent and scholarly address by Sir Michael Foster, to which we all listened with such deep interest, I feel that I do him no injustice when I recognize in it the practical application of the principles set forth by Mr. Herbert Spencer in his epoch-making essay on Education, or when I further recognize in it the spirit which animated the life-work of that man who, more than all others, I regard as the Nestor of dental education in England, Sir John Tomes.

I was told before I left America, and even since my arrival in England, that it would be quite useless to expect that anything which might be done as a result of the conferences of the Federation would have any effect in modifying existing views on the subject of dental education in Great Britain; and yet here in England there has been given out by one of her recognized educational authorities, and from one of her greatest universities, a statement of the principles of dental professional education the most liberal, logical, and reasonable which, in my judgment, has yet been uttered anywhere.

Like Mr. Spencer, Sir Michael Foster in his address recognizes the utilitarian character of professional knowledge, and the inevitable conclusion therefrom, that education should, from the beginning, be adapted to the uses which the knowledge thereby attained is intended to subserve. He has, by keeping that central idea in view, cut the Gordian knot which for years has confused our discussions and thought on the relationship of dentistry to medicine. His statement that the dentist, within the limits of his activities, is a "healer" places the dental practitioner upon the basis of a natural classification much more readily understandable than when he is regarded, either positively or negatively, as a medical specialist, for, lacking as we do an adequate definition of medicine, it is not yet possible to decide whether a dentist is a medical specialist or not.

The enthusiasm and unanimity of appreciation with which the address of Sir Michael Foster has been received clearly indicate the general acceptance which this representative international gathering has accorded to the views he has expressed. So evidently is that the case that it seems to me the further deliberations of this body may be most profitably confined to a study of the dental curriculum, or, in other words, to securing an arrangement of professional study in conformity with the principles set forth in the address, best suited to the education of the dentist. The best attainable curriculum is yet to be devised; the very fact that such marked differences are to be found in the curricula of dental colleges throughout the world is self-evident proof of the need for further investigation of the best methods for making dentists. It would simplify the question greatly if we should arrange all of the subjects now taught in all dental colleges into two categories: First, those which are essential, and, second, those which, though not essential, are desirable in the education of the dentist. We would then be in position, after having formulated a minimum essential curriculum, to provide for its continued expansion and improvement by the gradual inclusion of members of the category of desirables into that of the essentials.

Reference has been made to the importance of manual training as a feature of the dental curriculum, in order that there may be given to the student that high degree of manipulative dexterity without which he is unable to achieve success as a dental operator. We all admit the importance of manual training in dental education, but not all dental educators have clearly recognized an equally important consideration in that connection,—namely, the stage of development at which manual training should be undertaken by the student. I am not a physiologist, and I am glad to be in position to submit to the judgment of the distinguished physiologist, as well as educator, who is our presiding officer to-day whether it is not true that, in order to successfully train the hand to a high degree of dexterity, the manual education must be undertaken early in life; for a period quickly arrives in later years where such training becomes impossible. It was that fact which was clearly recognized by Sir John Tomes, and which he so energetically and practically advocated in his efforts at dental educational reform in this country.

We have frequent and familiar examples of an analogous state of affairs in connection with the use of the bicycle. It is quite possible for an individual after attaining adult years to learn to ride the wheel, but the later it is put off the more certainly does the unfortunate rider develop that anxious expression of countenance which in America we call the "bicycle face." The learner may in time know how to propel his machine, but in so doing he acquires infinitely more knowledge about every feature of the topography of the road-bed over which he travels, and never acquires that freedom and abandon begotten of the automatic muscular co-ordination with which the street urchin of a dozen years controls his machine. It is the necessity for manual training for the dental student in an early period of his career, when his muscular and nervous receptivity are at their maximum, that we find, in my judgment, the strongest argument in favor of a special curriculum for the dental student, and a sufficient reason why his special education should not be deferred until after he has pursued a standard course of medical training.

Professor Griffiths has referred to the importance of including the study of bacteriology in the dental curriculum. I know of no better illustration of the practical utility of a knowledge of baceriology to the operative dentist than that embodied in the statement recently made by Dr. Black, of Chicago, in which he said, with reference to the preparation of cavities in teeth preparatory to inserting fillings, that "the margins of all cavities should be laid down upon areas of tooth-structure which are relatively immune to the attacks of the bacteria which cause dental caries, in order to prevent a recurrence of the disease."

If that axiom be true, and I think that no one can successfully question its accuracy, the evident conclusion must be that no man can intelligently and successfully prepare a carious cavity in a tooth for filling except he be fortified by a fair knowledge of bacteriology. I feel that we may congratulate ourselves as dental teachers and practitioners upon our good fortune in securing the encouraging and far-sighted statement of dental educational principles embodied in the able address of our distinguished chairman and of those who have followed him.

*Sir Michael Foster* said, in answer to Dr. Kirk's inquiry, that for many years past he had urged that the education of the surgeon should not be delayed too long, because it was impossible after certain years to acquire that suppleness and dexterity of touch which were necessary for success. The mind grew old very slowly, and could be educated even late in life; but the body became old very soon, and it was necessary to train it while it was really young.

*Dr. Rosenthal* (Brussels) said that the evident conclusion of this conference was that nearly every one was of the same opinion, and he thought that Sir Michael Foster had said that morning nearly all that could be said on the matter. He proposed that Sir Michael's speech should be translated into all languages and sent to all the bodies in the world interested in the matter. In Belgium there was a movement towards putting dental education into the hands of the medical man, but the basis upon which such a resolution was taken was a monetary basis. The medical profession was so overcrowded that they thought specialization in dentistry would prove remunerative and relieve the overcrowding of their profession.

*Sir James Crichton Browne* was glad to have the opportunity of paying his tribute of admiration to the excellent address delivered that morning,—an address instinct with wit and wisdom, adorned by epigrams and similes which would not be easily forgotten. Sir Michael Foster referred to the early training of the dentist at the bench, and the subject had been further emphasized by the excellent observations of Dr. Kirk. Speaking from his own point of view, he attached great value and importance to the manual education of the dentist, and was inclined to attribute to that education a utility and significance that are not perhaps always generally recognized. Every surgeon knew that the movements of the hand were initiated in a certain group of centres of the middle region of the brain,—motor centres of the brain. But they were motor centres only in a special sense. They were not motor simply in the sense of sending forth impulses in response to excitations from without; they were motor in the sense of being the springs of movement, and they were receptacles in which was chronicled all the knowledge which the muscular operations put the man in possession of. The muscles not only obeyed the commands of the will, but they added infinitely to the information and intellectual acquisitions. The most cursory analysis of ideas revealed the fact that there were very few of them which were known purely by sensory impressions. The motor centres of the brain took an enormous share in mental life, and mental manifestations would be as impossible without them as would be the circulation of the blood without one ventricle of the heart. The highest possible functional activity of the motor centres was as important with a view to mental power as to muscular expertness, and the motor centres for the hand were very prominent among the motor centres of the brain. They were related to an organ which in its enormous combination of movements largely added to our intellectual resources, and it was evident that the highest possible functions of activity of those centres was of value in adding to intellectual grasp as well as adding to the expertness of the hand and to business success. But in order to have the highest possible functional activity of those centres it was necessary to have them trained betimes, and therefore it was necessary to give the student his manual training in dexterity very early in life; and by doing that one was not merely training the hand, but was helping to expand and develop the intellect.

*Dr. George Cunningham* thought the members would appreciate the results of the first general assembly in connection with the Federation, and he thought the discussion on education was a record which would be hard to beat. The Council had been authorized to appoint a Committee on Education, and he proposed, as a tribute to the success that had been met with that day, that the members should do something to promote the practical education of the public in dentistry. He proposed the appointment of a Committee on State Dental Service. It had been said at a meeting of the representative Board of the British Dental Association by a veteran whom he admired and respected for his past work, that he did not know what state dentistry was, and that he hated the word. State dentistry meant the utilization of the dentist's services by the state. The question of dentistry in the army never was settled. A war in Cuba was necessary to produce what was recommended before the war,—dentists for the army of the United States. Before the Boer war began there were dentists who as patriots thought their best place and their greatest assistance was in utilizing their services for the state, but their services were rejected. Four dentists had been sent out to the front to look after nearly a quarter of a million men,—very capable dentists, but without much experience. What were those few among so many? Their services would be lost, and the danger would occur that instead of giving the men the benefits of conservative dentistry there would be a greater utilization of the forceps, which to some had ceased to be a dental instrument at all. He would not be content with the dental services accorded to the army at the present moment by the War Department as long as the dentists were made servitors,—unless there was a superior mind of a dental character to guide the work. He therefore proposed the formation of a committee to take up the subject.

The proposition was seconded by Dr. Godon.

*Dr. J. Leon Williams* thought the greatest work which the Federation could do was not so much to educate the dentists as to educate the public. He therefore asked the Federation to keep in view two things,—first, the harmonizing of dental teaching, which meant to keep in very close touch with the most advanced scientific investigation. There must be always a divergence of opinion of all arts,—that was one of the conditions of progress,—but he felt there might be much more harmony in the application of scientific principles. A patient might go to one dentist who recommended a certain method of procedure, but if that patient went to another country, or to some one else for the same thing, a totally different course was recommended. In the advanced stage of scientific dentistry that was not necessary, and therefore he thought a part of the work of the Federation should be keeping in the closest possible touch with the great scientific questions and the harmonizing of their views. Perhaps even more important than that was an attempt to reach the public in some way. There had been a great international congress held in London on tuberculosis, and there was hardly an opinion held by advanced authority on that subject which had not been expressed. Medicine and surgery were in very much closer touch with the public than dentistry, and that closeness of touch was brought about very largely through the daily press, a thing which dentistry as a profession had almost neglected. There was a most woful amount of ignorance on the part of the public as to the possibilities of modern dentistry, and that was because the public was not in close touch with the dental profession and because the dental profession had not done its duty in educating the public in modern dentistry.

*Dr. Förberg* (Stockholm), as the hour was late, proposed that the subject should be discussed at the meeting to be held next year in Stockholm. He said he had taken the opportunity to give the general secretary the invitation for the Federation to meet in Stockholm, and he was also authorized to extend the invitation to the Executive Committee of the American Dental Society and the dental societies of Europe. Stockholm would do its best to welcome the Federation, but after the grand reception it had received in Cambridge, he was afraid the best would be exceedingly difficult.

*Sir Michael Foster* said the proposition of Dr. Förberg would be considered by the Council.

A vote of thanks was carried with acclamation to Sir Michael Foster, and the conference concluded.

After the conference in the afternoon a garden party was held at Merton Hall, at which a demonstration of long-off was given by Dr. Cunningham, Professor Sims Woodhead, and others, and the interval before the banquet was spent in an exceedingly pleasant manner.

[At the banquet in the evening, given at Downing College, Sir Michael Foster again occupied the chair.

There were the usual responses to toasts. These included remarks from Dr. Brophy, Sir Michael Foster, Dr. Godon, Dr. Harlan, Professor Sims Woodhead, Dr. Hessé, Mr. W. B. Patterson, Dr. Häderup, Sir James Crichton Browne, Dr. George Cunningham, and Dr. Sauvez. From the general tone of the remarks, there was great satisfaction felt at the success of the International Dental Federation, and a cordial feeling manifested for the kindly reception accorded the visiting delegates. Limited space will not permit the publication of a full report of this banquet.—Ed.]

(To be continued.)

